# Intelligent soft robotic gripper for non-destructive grasping and attribute recognition via multi-modal waveguide tactile sensors

**DOI:** 10.1038/s41378-026-01364-4

**Published:** 2026-06-15

**Authors:** Yanyun Fan, Chi Zhang, Yunheng Ying, Zhengang An, Qing Guo, Dachao Li, Lei Zhang

**Affiliations:** 1 State Key Laboratory of Precision Measuring Technology and Instruments, Tianjin University, Tianjin, 300072 China; 2 School of Electrical Engineering and Automation, Tianjin University of Technology, Tianjin, 300384 China

**Keywords:** Electrical and electronic engineering, Optics and photonics

## Abstract

The intelligent soft robotic gripper integrated with tactile sensors significantly enhances the robot’s execution capabilities in complex tasks, resolving critical shortcomings of traditional mechanical grippers—namely, fragile item breakage from rigid impacts, irregular object slippage, and inefficiency due to recognition errors. While electrical sensors *(e.g*., piezoresistive, capacitive) struggle with structural complexity, signal crosstalk, and environmental interference, optical waveguide tactile sensing offers superior sensitivity, rapid dynamics, and electromagnetic immunity. However, existing waveguide tactile systems face two key limitations: millimeter-scale waveguides cause beam divergence, limiting deformation sensitivity and complicating heterogeneous integration. Additionally, critical gaps remain in adaptive grasping control and contextual object recognition during manipulation. Herein, we present a soft robotic gripper integrated with slender elastic optical waveguide sensors (EOWS) and equipped with a closed-loop feedback control module to achieve intelligent grasping and object attribute recognition. The hand comprises three flexible silicone fingers, each finger seamlessly integrates three EOWS for multi-modal tactile sensing. These sensors exhibit high sensitivity to bending angle (0.273%/°), contact force (0.843%/N), and pressure (1.064%/N). Furthermore, a PID adaptive grasping control strategy and a long short-term memory (LSTM) deep learning algorithm are introduced to dynamically adjust the grasping force and intelligently recognize object attributes such as shape, size, and hardness, with accuracies exceeding 97% for each attribute. Ultimately, experimental validation via a smart fruit-sorting system highlights the platform’s potential for precision agriculture, intelligent logistics, and medical robotics, demonstrating robust, adaptive manipulation in real-world applications.

**Figure:**
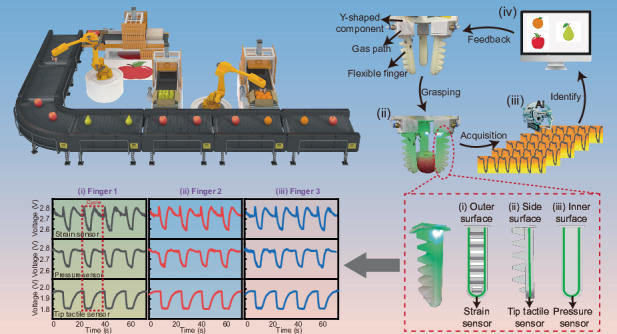
We present a soft robotic gripper seamlessly integrated with slender multi-modal elastic optical waveguide sensors (EOWS) and equipped with an adaptive control module to achieve intelligent grasping and object attribute recognition. Experimental validation via a smart fruit-sorting system highlights the platform’s potential for precision agriculture, intelligent logistics, and medical robotics, demonstrating robust, adaptive manipulation in real-world applications

We present a soft robotic gripper seamlessly integrated with slender multi-modal elastic optical waveguide sensors (EOWS) and equipped with an adaptive control module to achieve intelligent grasping and object attribute recognition. Experimental validation via a smart fruit-sorting system highlights the platform’s potential for precision agriculture, intelligent logistics, and medical robotics, demonstrating robust, adaptive manipulation in real-world applications

The intelligent soft robotic gripper represents a transformative advancement in robotic manipulation, integrating multi-modal sensors, biomimetic structural design, and high-precision force control algorithms to enable adaptive, non-destructive grasping in complex operational scenarios^1,2^. By synergizing tactile perception with cognitive decision-making, this system achieves intelligent recognition of object attributes—including type, size, shape, and hardness—with unprecedented accuracy. Such capabilities directly address critical industrial challenges: mitigating fragile item breakage caused by rigid collisions, preventing slippage of irregularly shaped payloads, and eliminating operational delays stemming from recognition errors^3,4^. Its multidimensional applications span precision agriculture (non-destructive fruit harvesting), logistics automation (heterogeneous package sorting), and medical robotics (surgical instrument manipulation), demonstrating transformative potential across diverse sectors^5–9^. The precise operation of intelligent soft robotic grippers relies on tactile sensors for the accurate capture, real-time feedback, and closed-loop control of multi-modal tactile information such as force and deformation.

Currently, the mainstream tactile sensing mechanisms mainly include capacitive, piezoresistive, piezoelectric, triboelectric, MEMS sensing, and vision sensing^10–18^. The performance comparison and applicable scenarios of various sensors are shown in Table S1. Among them, piezoresistive and capacitive sensors have mature manufacturing processes, but usually require complex structural designs to achieve multi-modal sensing and are easily affected by signal crosstalk. Piezoelectric and triboelectric sensors only respond to dynamic contact signals and are difficult to achieve stable static force measurement. MEMS sensors offer high precision and stability, but their high stiffness and poor compatibility prevent conformal integration with soft grippers. Vision sensing provides rich contact morphology information, but it has a large volume, limited real-time performance, and susceptibility to lighting and occlusion. In contrast, the optical waveguide type features high sensitivity, rapid response, electromagnetic interference immunity, low hysteresis, and excellent multi-modal sensing capabilities^19–23^. It can achieve high integration and distributed deployment with soft grippers, and is more suitable for practical application needs such as non-destructive grasping and flexible interaction. Bai et al. ^24^ proposed a dual-core elastic optical fiber system that differentiates bending, stretching, and local pressure by monitoring color and intensity changes, detecting forces during finger joint deformation. Zhao et al. ^25^ developed a stretchable optical waveguide as a tactile feedback nerve integrated into a prosthetic hand, achieving active perception of object shape and softness. Existing research has made significant progress in active tactile perception but still has limitations: the millimeter-scale diameter of optical waveguide sensors results in beam divergence, thereby restricting their sensitivity to minute deformations, and the integration of heterogeneous components within soft robotic grippers poses significant challenges. Additionally, few studies have focused on adaptive control during grasping and intelligent recognition of object attributes such as shape, size, and hardness.

In this study, we present a soft robotic gripper integrated with slender elastic optical waveguide sensors (EOWS) and equipped with an adaptive control module to achieve intelligent grasping and object attribute recognition. The hand consists of three flexible silicone fingers, each finger seamlessly integrated with three EOWS. The EOWS exhibit remarkable stretchability exceeding 400% and demonstrate excellent sensitivity to the bend, contact force, and pressure, with sensitivities of 0.0073 μA/°, 0.0225 μA/N, and 0.0284 μA/N, respectively. By integrating an adaptive grasping control strategy and long short-term memory (LSTM) deep learning algorithm, the intelligent soft robotic gripper can dynamically adjust the grasping force and intelligently recognize object shape, size, and hardness. Finally, the system demonstrates intelligent sorting of various types of fruit, providing solutions for the development of industrial non-destructive grasping technologies.

## Experimental section

### Materials

The PU fiber substrate (diameter: 0.5 mm, refractive index: 1.52) was purchased from Shengyi Plastic Insulation Materials *Co., Ltd*., China. PDMS was purchased from Chemart Chemical Technology *Co., Ltd*., Tianjin, China. The LED microchip (0.5 mm*0.5 mm) was purchased from Shengyuan Electronics *Co., Ltd*., Shenzhen, China. The photodiode (LSSPD-1.2-2P-03) was purchased from Minguang Technology *Co., Ltd*., Beijing, China. The T-shaped plug was obtained from Poroclor *Co., Ltd*., Shenzhen, China. The UV ultraviolet light-curing adhesive (UV-20) was purchased from Yinghuoban Technology *Co., Ltd*., Wenzhou, China.

### Fabrication of EOWS

First, PU elastic fibers were ultrasonically cleaned in anhydrous ethanol and deionized water sequentially, and then dried for later use. Then, a low-refractive-index PDMS solution was prepared at a 20:1 precursor-to-curing-agent ratio and vacuum-degassed. Next, the cleaned PU fibers were dip-coated in the PDMS solution, vertically placed for 1 h to ensure uniform cladding flow, and then dried at 60°C for 2 h to achieve complete PDMS cladding curing. Finally, one end of the PU/PDMS waveguide was coupled to an LED microchip with UV-curable adhesive, and the other end was connected to a photodiode via a T-shaped plug.

### Fabrication of the intelligent soft robotic gripper

**Fig. 1:**
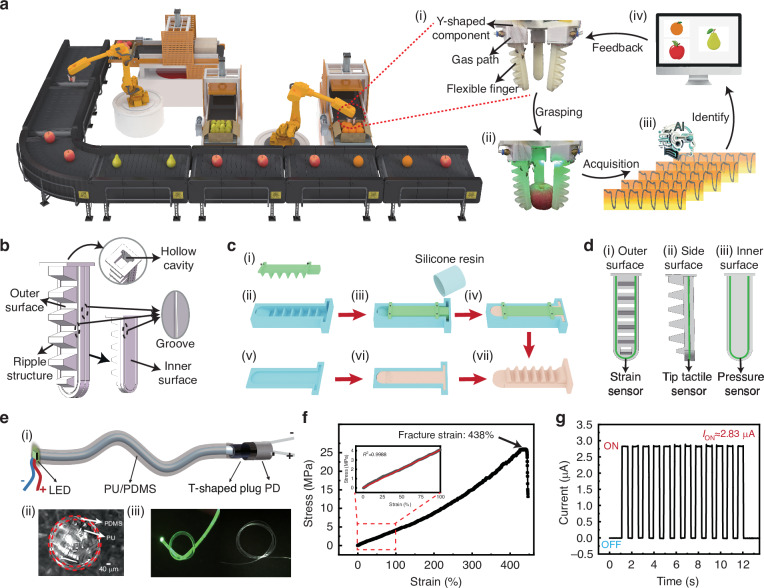
**Intelligent soft robotic gripper configuration**. **a** Conceptual diagram illustrating the intelligent grasping system of soft robotic grippers for non-destructive grasping and attribute recognition of fruits, irregularly shaped packages, and hazardous materials. (i) soft robotic gripper structure, (ii) multi-modal tactile signal collection, (iii) LSTM deep learning algorithm, and (iv) intelligent recognition display. **b** Structural design of the flexible finger. (i-ii) mold for the outer surface of flexible finger, (iii) pour silicone into the mold, (iv) cure at room temperature, (v) mold for the inner surface of flexible finger, (vi) pour silicone into the mold and cure, (vii) bond with silicone adhesive after demolding. **c** Fabrication process of the flexible finger. **d** EOWS configurations of the flexible finger on the (i) outer surface, (ii) side surface, and (iii) inner surface for the detection of bending angle, contact force, and pressure, respectively. **e** (i) structural design, (ii) cross-sectional optical micrograph, and (iii) knotted and coiled states of the EOWS. **f** Stress-strain curve of the EOWS, exhibiting a stretchability exceeding 400%. **g** On-off electrical signal response of the EOWS under 3 V DC actuation

First, 3D-printed finger molds (Fig. 1c) are poured with silicone into the molds, vacuum-degassed, and naturally cured for 24 h before demolding. Then, the upper and lower sections were bonded with silicone adhesive to form flexible fingers with hollow pneumatic chambers. Next, the diameter of 500 μm of EOWS was embedded into grooves on the finger’s outer, side, and inner surfaces using tweezers, encapsulated with silicone, vacuum-degassed, and cured. Finally, three flexible fingers were fixed at 120° intervals on a Y-shaped bracket to assemble the intelligent soft robotic gripper.

### Characterization and electrical measurements

The periodic strain was provided by linear motors (Linmot E1100, LinMot, *Inc*., Elkhorn, WI, USA). The constant pressure value was provided through the Vernier double-range force sensor (Vernier Science Education, Beaverton, OR, USA). The electrical performance was measured by a programmable electrometer (Keithley 6514, Keithley Instruments, Cleveland, OH, USA). Data was recorded by software written in LabVIEW. Multi-channel data was measured through the data acquisition unit (Keithley DAQ6510, Keithley Instruments, Cleveland, OH, USA).

### I-V transimpedance amplifier conversion circuit

As shown in Figure S13, the I-V transimpedance amplifier conversion circuit board is equipped with an AD825 operational amplifier and an LF353 operational amplifier. With a maximum gain of 1.2 × 10^7^, this circuit enables the conversion and amplification of weak current-to-voltage signals, facilitating efficient data acquisition by the DAQ6510.

### LSTM algorithm for intelligent attributes recognition

LSTM algorithm data set configuration: 1) Data collection: A total of 16 objects were included, with each object being grasped at least 200 times, each lasting 5 s. 2) Training and testing: Of the samples in the data set, 80% were used for training, while the remaining 20% were used for testing. To ensure the stability and reliability of the model, the S-fold cross-validation method was employed, and the training set (80%) was randomly divided into five parts. To prevent overfitting of the model, the ADAM optimizer and L2 regularization were utilized for optimization. This data set facilitates accurate classification and recognition of object attributes.

## Results and discussion

### Intelligent soft robotic gripper configuration

For smart non-destructive grasping, the intelligent soft robotic gripper needs to have the ability to perceive, recognize, and provide feedback on multi-modal tactile signals. In this context, the intelligent soft robotic gripper system we design consists of four main components: a flexible soft robotic gripper, waveguide-based tactile sensors, a PID grasping controller, and an LSTM recognition algorithm, as shown in Fig. 1a. First, the soft robotic gripper employs a three-finger silicone flexible design, which provides excellent grasping stability compared to the two-finger design and adaptive wrapping of objects, effectively reducing the risk of grasping damage. Second, the waveguide tactile sensors—whose features include high sensitivity, fast response, anti-electromagnetic interference, and high stretchability—are arranged on the outer, side, and inner surfaces of the flexible fingers, which enables the capture of multi-modal tactile signals during the grasping process. Then, the PID control is utilized to regulate the air pressure of the pneumatic drive unit, precisely controlling the inflation (grasping), pressure holding (holding), and deflation (releasing) processes of the soft robotic gripper’s pneumatic chambers. Finally, the LSTM deep learning algorithm is employed to intelligently recognize the attributes of the grasped objects, including shape, size, and hardness. The recognition results are then fed back to the soft robotic gripper for adaptive adjustments, thereby ultimately achieving intelligent non-destructive grasping.

The structural design of the flexible finger is shown in Fig. 1b, with its outer surface featuring a corrugated deformable layer that exhibits low hardness and high stretchability, enabling significant deformation upon inflation. The inner surface is a flat constraint layer designed to suppress excessive deformation of the outer surface. When the chamber is inflated, the increased internal pressure creates a pressure difference between the inner and outer layers, driving the finger to bend towards the side of the constraint layer, thereby achieving a grasping action (Figure S1). As shown in Fig. 1c, the finger is fabricated using mold casting, with the main material being the flexible silicone Dragon Skin 30, which offers both excellent flexibility and biocompatibility. To measure the bending angle, contact force, and pressure during the grasping process, elastic optical waveguide sensors (EOWS) are integrated on the outer surface, side surface, and inner surface of the flexible finger, respectively, as shown in Fig. 1d (Figure S2). The EOWS consists of three parts: a micro-LED patch, a flexible PU/PDMS optical waveguide fiber, and a photodiode (PD), As shown in Fig. 1e. The PU/PDMS waveguide features elastic PU fibers as the core (refractive index 1.52) and PDMS elastomer as the cladding (refractive index 1.417). The substantial refractive index difference gives the waveguide a numerical aperture (NA) of 0.55, promoting total internal reflection for efficient light guidance (Figure S3). Moreover, the PU/PDMS optical waveguide exhibits excellent stretchability, with a strain capacity exceeding 400%, as shown in Fig. 1f. Various colored lights can be stably transmitted within the EOWS without noticeable scattering or loss phenomena (Figure S4). Even when bent to a 2 mm radius, the waveguide maintains bright, stable light output, demonstrating exceptional flexibility and optical transmission characteristics. A 3 V DC voltage on the LEDs produces green light that transmits efficiently through the PU/PDMS waveguide to the PD, generating a current signal. As shown in Fig. 1g, the EOWS also shows fast response and stability in repeated on-off cycles (Figure S5).

### Tactile sensing characterization of the EOWS

**Fig. 2:**
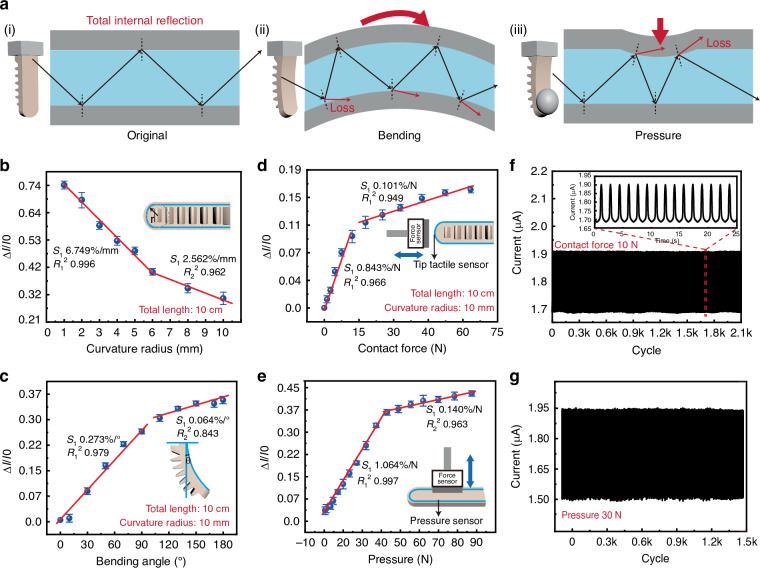
**Tactile sensing characterization of the EOWS**. **a** Sensing mechanisms of the EOWS for (i) original, (ii) bending, and (iii) pressure conditions. **b** Response of the EOWS to different curvature radii ranging from 1 mm to 10 mm. **c** Response of the EOWS to different bending angles ranging from 0° to 180° at a curvature radius of 10 mm. **d** Response of the EOWS to different contact forces ranging from 0 to 65 N at a curvature radius of 10 mm and a bending angle of 0°. **e** Response of the EOWS to different pressures ranging from 0 to 90 N at a curvature radius of 10 mm and a bending angle of 0°. **f** Stability of the EOWS at a curvature radius of 10 mm, a bending angle of 0°, and a contact force of 10 N. **g** Stability of the EOWS at a curvature radius of 10 mm, a bending angle of 0°, and a pressure of 30 N

Figure 2a shows the EOWS sensing mechanism. Under mechanical stimuli such as strain, bending, or pressure, the sensor undergoes controlled deformation (Figure S6), causing axial deviation of the light-guiding path and systematic variation of the incident angle. According to the principle of total internal reflection, when the incident angle is less than the critical angle, some light can’t be totally within the waveguide and escapes, leading to light loss. Therefore, by detecting the loss current variation caused by this light loss, high-sensitivity sensing of the mechanical stimuli can be achieved.

In the design of the soft robotic gripper, the curvature radius of the EOWS on the surface of the flexible fingers plays a crucial role in determining the sensing performance. As shown in Fig. 2b, the sensitivity reaches 6.749 %/mm when the radius of curvature is 1 mm-6 mm, and decreases to 2.562 %/mm when it is 6 mm-10 mm. As the curvature radius decreases, the optical transmission loss gradually intensifies, which is detrimental to the detection of multi-modal tactile information. Taking into account the dimensional design of the flexible fingers and the optimization requirements of the sensor performance, a curvature radius of 10 mm is ultimately selected. Subsequently, the characterization of multi-modal tactile sensing information of flexible fingers was carried out, including bending, contact force, and pressure. As shown in Fig. 2c, the sensitivity is 0.273%/° for bending angles of 0°-110°, and drops to 0.064%/° for 110°-180°, possibly due to complex stress distribution in the waveguide material and light transmission mode changes at high curvature. Contact and grasping forces are applied to the EOWS through the linear motor and pressure sensor (Figures S7–S8). As shown in Fig. 2d, the contact force sensitivity is 0.843%/N below 15 N, and 0.101%/N above 15 N. As shown in Fig. 2e, the pressure sensitivity is 1.064%/N below 45 N, and 0.140%/N above 45 N, likely because EOWS deformation is limited at high forces. When loading unloading different pressures on EOWS, the hysteresis coefficient is only 1.30% (Figure S9). After being converted by an I-V transimpedance amplifier, the output voltage shows a good linear relationship with pressure (Figure S10). As shown in Figs. 2f and 2g, cyclic tests are conducted under a contact force of 10 N and a pressure of 30 N, respectively. No significant drift or fluctuation is observed, indicating the excellent stability of the EOWS. When the temperature is within the range of 24–50 °C, the output of EOWS stabilizes at 2.590 ~ 2.601 μA, with a maximum deviation rate of only 0.32% relative to room temperature (24 °C), and the measurement standard deviation at all temperatures is below 0.041 μA, meeting the requirements for daily use (Figure S11). In addition, by constructing a multivariate decoupling scheme (Figure S12 (a)), the outer surface pure bending data is used as the benchmark to perform real-time compensation for the inner and side surface EOWS, achieving the extraction of bending angle, pressure, and contact force information. During the empty grasping operation of the soft robotic gripper (Figure S12(b)), the standard deviation of the output voltage of the outer, side, and inner surface sensors is only 0.334%, 0.612%, and 0.553%, indicating that bending has a consistent effect on all sensors, providing a basis for quantitative decoupling. Therefore, the EOWS demonstrates reliable multi-modal tactile sensing capabilities (bending, contact force, and pressure) and superior stability, which provide a solid foundation for its practical application in soft robotic grippers.

### Closed-loop feedback grasping control of intelligent soft robotic gripper

**Fig. 3:**
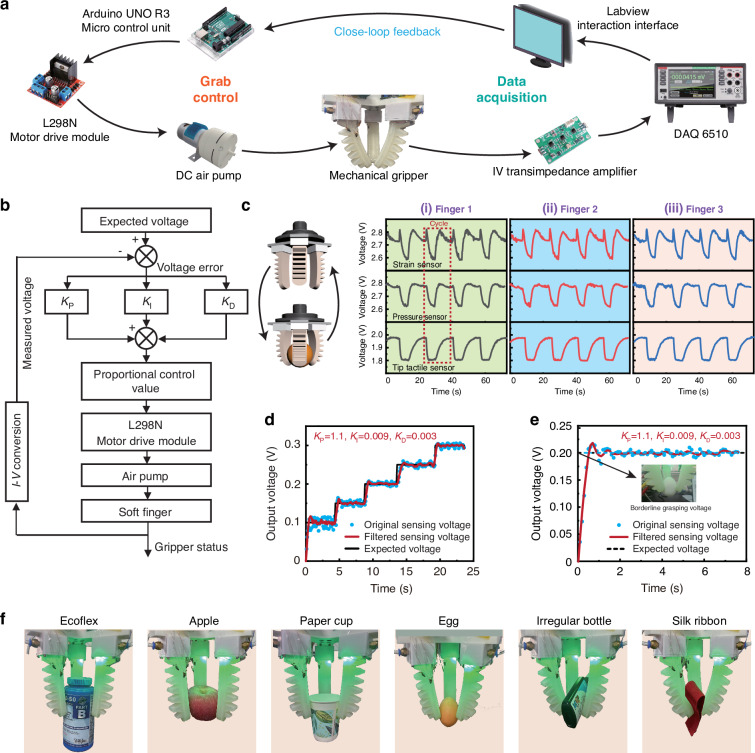
**Closed-loop feedback grasping control of intelligent soft robotic gripper**. **a** The closed-loop closed-loop feedback grasping control system architecture, which includes the pneumatic actuation module, data acquisition module, and PID controller. **b** Schematic diagram of the PID closed-loop feedback grasping control logic. **c** During multiple grasps of a 5 cm sphere, the multi-modal tactile voltage signals output by the EOWS are used for PID parameter tuning, with the optimal parameters ultimately determined as *K*_P_ = 1.1, *K*_I_ = 0.009, and *K*_D_ = 0.003. **d** Under the optimal PID parameters, the step response curve of PID controller. **e** The voltage response curve of the soft robotic gripper during adaptive grasping of an egg (at critical grasping force. **f** Demonstration of the adaptive grasping performance of the intelligent soft robotic gripper on various objects

To achieve closed-loop feedback grasping control of the intelligent soft robotic gripper, its control system mainly consists of a pneumatic control module, a data acquisition module, and a proportional-integral-derivative (PID) control module. The overall architecture is shown in Fig. 3a. The pneumatic control module utilizes an Arduino UNO R3 microcontroller to generate pulse width modulation (PWM) signals, driving the L298N motor driver to regulate the power input of a DC air pump. This enables continuous stepless adjustment of air pressure, precisely controlling the inflation, holding, and deflation actions of the flexible fingers to adapt to grasping and releasing various objects. The data acquisition module employs an I-V transimpedance amplifier (circuit diagram illustrated in Figure S13) to convert weak current signals from EOWS into voltage signals, which are then acquired by a DAQ6510 device. This configuration achieves preliminary signal amplification and effectively suppresses background noise, improving the signal-to-noise ratio. The PID control module dynamically adjusts the microcontroller’s output signal based on real-time voltage feedback from the inner surface EOWS, forming a closed-loop control loop for grasping force to achieve closed-loop feedback grasping of different objects.

As shown in Fig. 3b, an closed-loop feedback grasping logic framework for an intelligent soft robotic gripper based on PID control is constructed, with the core focus on the determination of proportional, integral, and derivative parameters (*K*_P_, *K*_I_, *K*_D_). Figure 3c shows the output voltage signals of 9 EOWS during multiple grasps of the same object by the bionic dexterous hand, which are used for PID parameter tuning. Based on the general principles of PID tuning^26–28^ and through multiple experimental optimizations (Figure S14), the optimal parameters are determined to be *K*_P_ = 1.1, *K*_I_ = 0.009, and *K*_D_ = 0.003. Figures 3d, 3e show the step-wise increase and tracking process of the sensor output voltage. The PID controller can precisely and rapidly adjust the voltage of the air pump proportional control valve to achieve the target voltage fed back by the EOWS, thereby effectively supporting the intelligent soft robotic gripper to achieve closed-loop feedback grasping of different objects. Under PID closed-loop control, the intelligent soft robotic gripper can stably reach this critical voltage within 0.71 s, with a steady-state error of 2.12%. The soft robotic gripper can also control the system to increase the expected voltage to increase air pressure and enhance gripping force when an object slip (Figure S15). If the output of the pressure sensor drops too quickly, the system will adjust the expected voltage of the PID controller to zero to prevent over-inflation and damage, and then readjust the expected voltage and perform grasping again. In addition, the soft robotic gripper continuously grasped for 10 h, and the output voltage of the inner sensor stays stable within 2.48–2.70 V, maintained good stability (Figure S16). Figure 3f further demonstrates that the intelligent soft robotic gripper system is capable of stably and rapidly grasping various objects, providing technical support for the application of robotic technology in the field of precision operations.

### Intelligent attributes recognition algorithm via LSTM deep learning

**Fig. 4:**
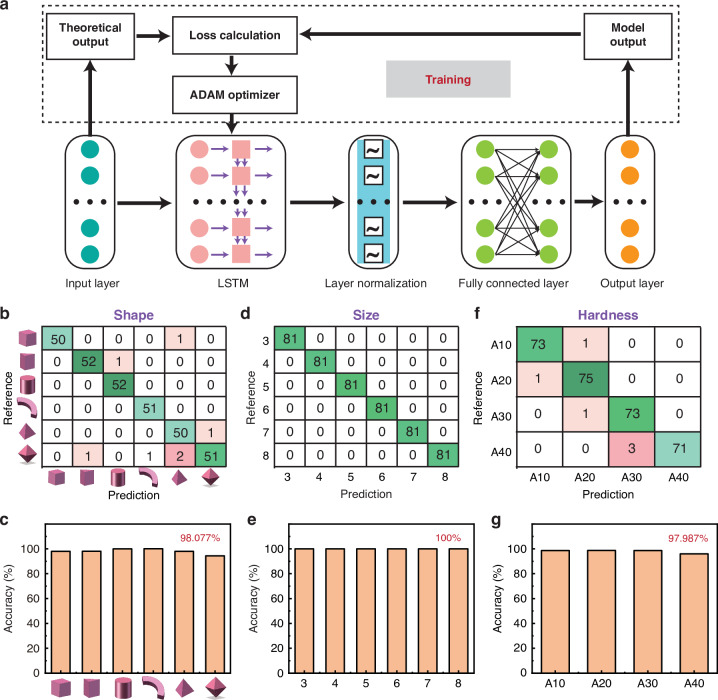
**Intelligent attributes recognition algorithm via LSTM deep learning**. **a** Network architecture of the LSTM algorithm. **b**, **c** Confusion matrix and recognition accuracy for 6 different shapes using the LSTM algorithm, resulting in an average accuracy of 98.08% (Fold 3). **d**, **e** Confusion matrix and recognition accuracy for 6 different sizes of spheres using the LSTM algorithm, resulting in an average accuracy of 100%. **f, g** Confusion matrix and recognition accuracy for 6 different hardness levels of Shore blocks using the LSTM algorithm, resulting in an average accuracy of 97.99% (Fold 2)

In order to intelligently identify the attributes of object size, shape, and hardness by analyzing the multidimensional time-series data during grasping, the long short-term memory (LSTM) deep learning algorithm is introduced. It can store intermediate-state info over long time intervals and capture long-term dependencies in time-series data, making it particularly suitable for complex tasks requiring contextual state associations^29–31^. As shown in Fig. 4a, the network has three layers: the input layer connects to the signals from 9 sensors, the LSTM hidden layer has 256 memory units, and the output layer generates (*n* + 1) type discriminative results (n object labels and an empty grasp state). The data set includes three feature categories: shape discrimination (cube, triangular prism, cylinder, circular ring, triangular pyramid, and octahedron), size recognition (3–8 cm diameter spheres), and hardness identification (10–40 A shore hardness blocks). During data collection, the intelligent soft robotic gripper vertically grasps objects at fixed positions, with each grasp lasting 5 s. For 16 feature objects, 200 repeated grasps per type are performed, collecting over 3200 valid data sets, providing a reliable training foundation for LSTM-based feature recognition research.

This study use stratified 5-fold cross-validation to randomly divide the original data set into five subsets to ensure the stability and reliability of different subsets. The ADAM optimizer is used to avoid local minima and oscillation during training. L2 regularization prevents overfitting, and layer normalization improves model accuracy. This ensures model stability in complex data environments. The average recognition accuracy and standard deviation of object shape, size, and hardness are 97.891% ± 0.114%, 100.00% ± 0.00%, and 97.740% ± 0.047%, respectively. Figure 4b–g show the optimal training efficiency curves (Figure S17), the confusion matrix, and recognition accuracy for object shape, size, and hardness. The recognition accuracy for each fold is shown in Table S2. In addition, we also demonstrated the soft robotic gripper’s ability to grasp and recognize objects from different directions. The soft robotic gripper captures five types of fruits, mango, peach, strawberry, orange, and tomato, from three different angles and constructs a data set. The recognition accuracy of fruit types still reaches 99.012% ± 0.123% (Figure S18). The performance comparison of soft robotic gripper sensors with different mechanisms is shown in Table S3, indicating that the sensors in this study have comprehensive advantages in multi-modal tactile information detection (pressure, bending, and contact force) and intelligent recognition ability (shape, size, and hardness). Overall, the recognition algorithm based on LSTM exhibits stable object attribute recognition ability under current testing conditions, providing preliminary conceptual validation for the subsequent research of the intelligent soft robotic grippers in industrial assembly, agricultural harvesting, and logistics sorting applications.

### Application of the intelligent soft robotic gripper in the smart fruits sorting system

**Fig. 5:**
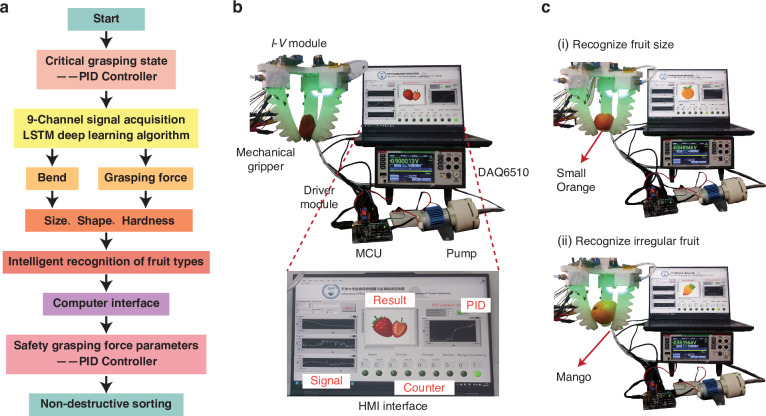
**Application of intelligent soft robotic gripper in smart sorting system**. **a** Workflow diagram of the smart fruits sorting system via the intelligent soft robotic gripper. **b** Demonstration of non-destructive fruit grasping and species recognition based on the intelligent soft robotic gripper system. **c** Demonstration of intelligent recognition of fruit sizes and irregular shapes

We demonstrate the application of the intelligent soft robotic gripper in a smart fruits sorting system (SFSS). The workflow of the SFSS via intelligent soft robotic gripper is illustrated in Fig. 5a: Firstly, the soft robotic gripper makes contact with the target fruit and reaches a critical grasping state, while the EOWS are employed to collect multi-modal tactile signals. Then, the grasping force is adaptively regulated through PID controller based on the analysis of the multi-modal tactile information. Meanwhile, the attributes of the fruit, including shape, size, and hardness, are intelligently recognized using the LSTM algorithm, and the type of fruit is displayed on the computer interface. Finally, the system invokes the preset safe clamping force parameters for the specific type of fruit to achieve non-destructive grasping. As shown in Figs. 5b, 5c, the intelligent soft robotic gripper is capable of successfully smart sorting irregularly shaped fruits such as strawberries, mangoes, and bananas. For fruits with similar shapes, such as apples, tomatoes, and oranges, the system can intelligently recognize the species and further sort them based on the preset size criteria. In summary, the intelligent soft robotic gripper system has realized intelligent fruit sorting based on multi-modal tactile perception and intelligent attribute recognition algorithm, demonstrating broad application prospects in the field of intelligent non-destructive sorting of agricultural products, fragile items, and hazardous materials.

## Conclusions

In this work, we present a soft robotic gripper integrated with slender elastic optical waveguide sensors (EOWS) and equipped with an adaptive control module to achieve intelligent grasping and object attribute recognition. The intelligent soft robotic gripper comprises three flexible fingers, each seamlessly integrated with three EOWS units, enabling real-time acquisition of multi-modal tactile signals during object interactions for precise grasping control. The EOWS exhibit exceptional sensitivity to bending angle (0.273%/°), contact force (0.843%/N), and pressure (1.064%/N). By introducing adaptive grasping control and the LSTM algorithm, the intelligent soft robotic gripper can dynamically adjust the grasping force, recognize object attributes, including shape, size, and hardness, with accuracies exceeding 97% for each attribute. In addition, the developed smart sorting system based on an intelligent soft robotic gripper successfully executes non-destructive grasping and classification of diverse fruits, providing a new path for intelligent grasping in industrial assembly, agricultural picking, logistics sorting, and hazardous material handling.

Although the intelligent soft robotic gripper integrated with EOWS has been successfully developed to achieve multi-modal tactile sensing, closed-loop grasping control, and object attribute recognition. But there is still room for optimization and extension to enhance its performance in complex real-world scenarios. Future work will focus on the following aspects to further improve the robustness, generalization ability, and engineering applicability of the system:

1. Although the bending data from the outer surface EOWS can be used to compensate for the coupled signals of the inner and side sensors in real time, it is necessary to combine advanced machine learning algorithms to realize high-precision mathematical decoupling of bending, contact force, and pressure signals, further improving the stability and accuracy of sensing signals.
2. At present, target objects can be recognized by updating the object database and iterating the model incrementally. In the future, transfer learning, few-shot learning, or meta-learning strategies can be introduced to expand the diversity and coverage of training samples and improve the system’s generalization to unseen objects.
3. The sensing system can operate continuously for a long time under conventional environments. However, its reliability under extreme conditions such as high humidity, chemical corrosion, and strong electromagnetic interference still needs to be further optimized.
4. The sensing system can be integrated with different types of robotic arms and equipped with visual sensors to enhance environmental perception, further improving the adaptability of the soft robotic gripper under complex working conditions.

## Supplementary information

Intelligent Soft Robotic Gripper for Non-destructive Grasping and Attribute Recognition via Multi-modal Waveguide Tactile Sensors

Application of the Intelligent Soft Robotic Gripper in Smart Fruits Sorting System

## Data Availability

The data that support the findings of this study are available from the corresponding author upon reasonable request.
