# Supplementary material for: Intelligent soft robotic gripper for non-destructive grasping and attribute recognition via multi-modal waveguide tactile sensors
Source: Microsyst Nanoeng. 2026 Jun 15;12:233. doi: 10.1038/s41378-026-01364-4 (PMC13265845; doi:10.1038/s41378-026-01364-4)
Supplement: Supplementary file 1 — Intelligent Soft Robotic Gripper for Non-destructive Grasping and Attribute Recognition via Multi-modal Waveguide Tactile Sensors [file 41378_2026_1364_MOESM1_ESM.docx]

**Supporting Information**

**Intelligent Soft Robotic Gripper for Non-destructive Grasping and Attribute Recognition via Multi-modal Waveguide Tactile Sensors**

Yanyun Fan^1, #^, Chi Zhang^1, 2, #^, Yunheng Ying^1, #^, Zhengang An^1^, Qing Guo^1, *^, Dachao Li^1,*^, Lei Zhang^1, *^

^#^ Yanyun Fan, Chi Zhang and Yunheng Ying contributed equally to this work.

^1^ State Key Laboratory of Precision Measuring Technology and Instruments, Tianjin University, Tianjin, 300072, China.

^2^ School of Electrical Engineering and Automation, Tianjin University of Technology, Tianjin 300384, China.

*Corresponding author. Qing Guo, E-mail: guoq@tju.edu.cn; Dachao Li, E-mail: dchli@tju.edu.cn; Lei Zhang, E-mail: zhangleitd@tju.edu.cn

Content of the SupplementaryInformation

**Figure S1** Physical pictures of flexible fingers and schematic diagrams of inflatable bending.

**Figure S2** (a) Physical image of the flexible finger integrated with EOWS. (b) Schematic diagram of the relationship between arc length and bending radius when a flexible finger is bent.

**Figure S3** (a) Microscopic optical images of the surface of a PU core with a diameter of 500 μm. (b) Photoconductive photograph of green light within the PU fiber core.

**Figure S4** (a) The light conduction phenomenon in EOWS. (b) The propagation of light of different colors in EOWS over 30 cm.

**Figure S5** The switching response time of EOWS.

**Figure S6** (a) The sensing mechanism of strain by EOWS. (b) Output curves of EOWS under different strain degrees. (c) Output current loss of EOWS and unencapsulated PU fibers at 0-30% strain. (d) Stability curve of EOWS at 20% strain.

**Figure S7** (a) The contact force curve applied to the tip of the EOWS. (b) The output current curve of EOWS during the application of contact force and its corresponding stress.

**Figure S8** The output curves of EOWS under different pressures.

**Figure S9** (a) Output current curve of EOWS at different pressures. (b) Hysteresis curves of EOWS under different pressures.

**Figure S10** (a) The output voltage curve of the inner surface EOWS under different pressures. (b) Voltage response of the inner surface EOWS under pressure of 0-22 N.

**Figure S11** (a) Current response of the EOWS under 24-50°C. (b) Mean value and standard deviation of the steady-state current.

**Figure S12** (a) Schematic of the multivariable decoupling algorithm based on bending signal compensation. (b) The output response of the sensor in the empty grasping state.

**Figure S13** Schematic diagram of 3-channel I-V transimpedance amplifier.

**Figure S14** Adjust the voltage response curves of KP, KI and KD under no-load conditions.

**Figure S15** Voltage response of soft robotic gripper in slip detection.

**Figure S16** Output stability of the soft robotic gripper during 10 h of continuous operation.

**Figure S17** The training efficiency curve of bio-inspired dexterous hands in recognizing the size, shape and hardness of objects.

**Figure S18** (a) Soft robotic gripper grabs each fruit from different angles. (b-d) Training efficiency curve, confusion matrix, and recognition accuracy of soft robotic gripper recognition of fruit types.

**Table S1** Comparison of the characteristics of different tactile sensing modalities.

**Table S2** The recognition accuracy rate of shape, size and hardness based on 5-fold cross-validation.

**Table S3** Performance of different sensors integrated into the soft robotic gripper.


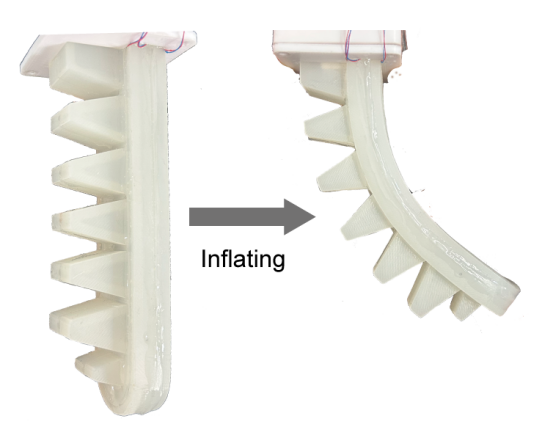


**Figure S1** Physical pictures of flexible fingers and schematic diagrams of inflatable bending.


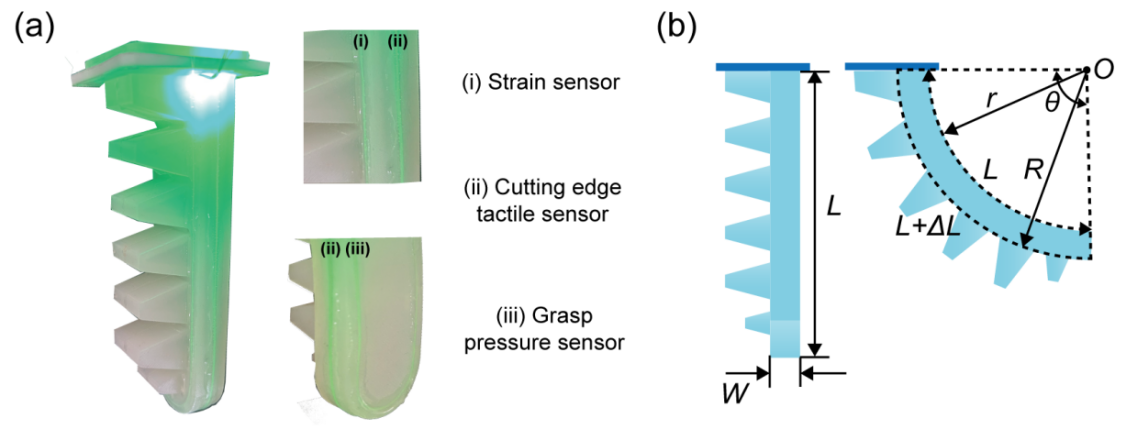


**Figure S2** (a) Physical image of the flexible finger integrated with EOWS. (b) Schematic diagram of the relationship between arc length and bending radius when a flexible finger is bent (*L* represents the length of the flexible finger, *W* represents the thickness, *ΔL* represents the change in length, *r* represents the radius of curvature of the inner sensor, *R* represents the radius of curvature of the outer sensor, and *θ* represents the central Angle).


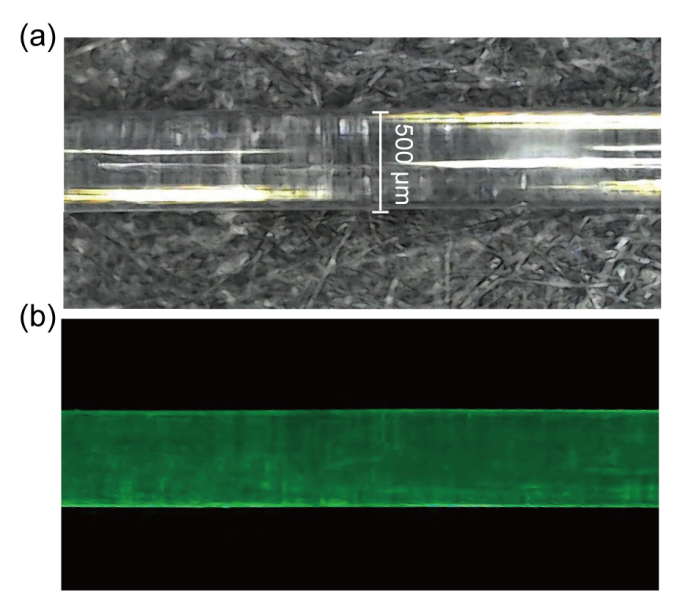


**Figure S3** (a) Microscopic optical images of the surface of a PU core with a diameter of 500 μm. (b) Photoconductive photograph of green light within the PU fiber core.


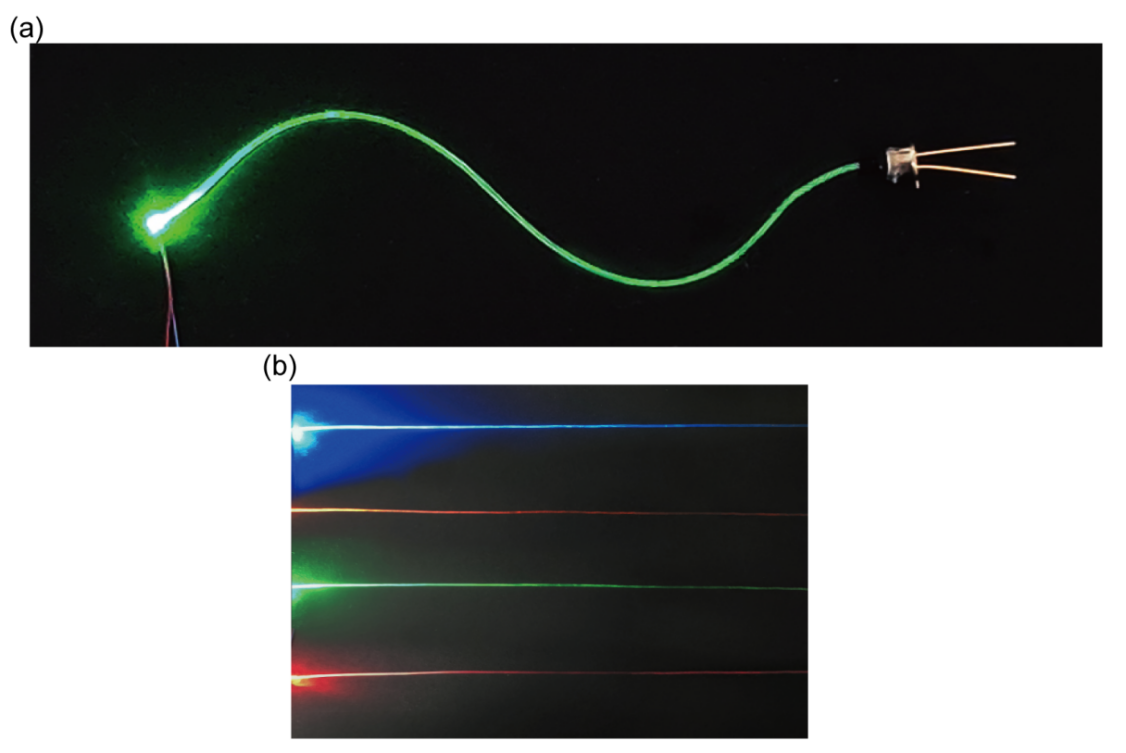


**Figure S4** (a) The light conduction phenomenon in EOWS. (b) The propagation of light of different colors in EOWS over 30 cm.


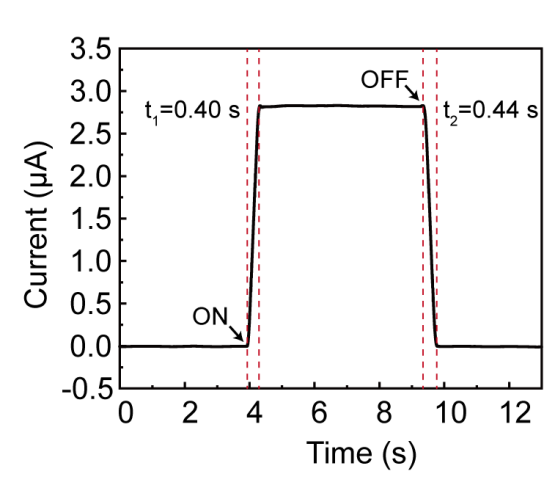


**Figure S5** The switching response time of EOWS.


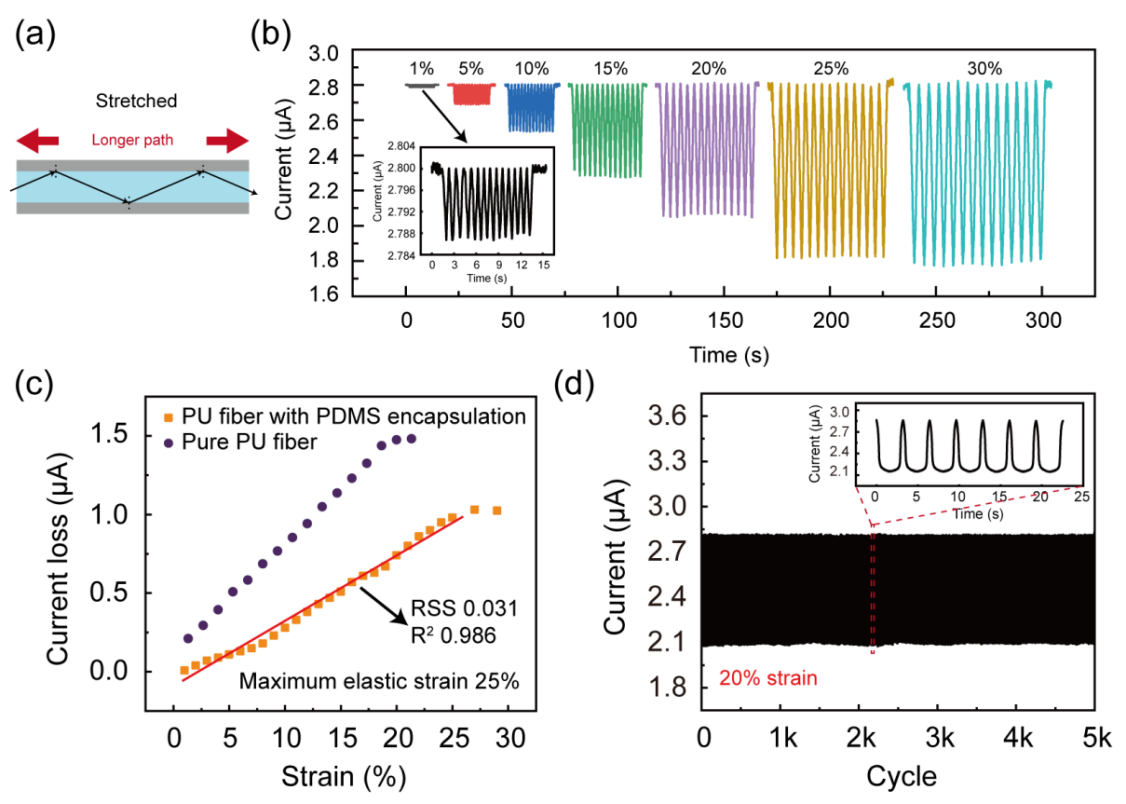


**Figure S6** (a) The sensing mechanism of strain by EOWS. (b) Output curves of EOWS under different strain degrees. (c) Output current loss of EOWS and unencapsulated PU fibers at 0-30% strain. (d) Stability curve of EOWS at 20% strain.


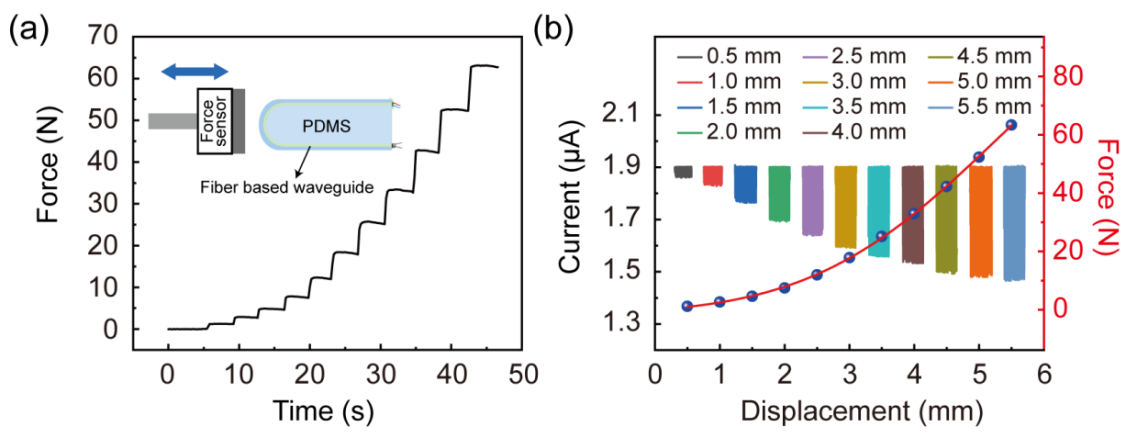


**Figure S7** (a) The contact force curve applied to the tip of the EOWS. (b) The output current curve of EOWS during the application of contact force and its corresponding stress.


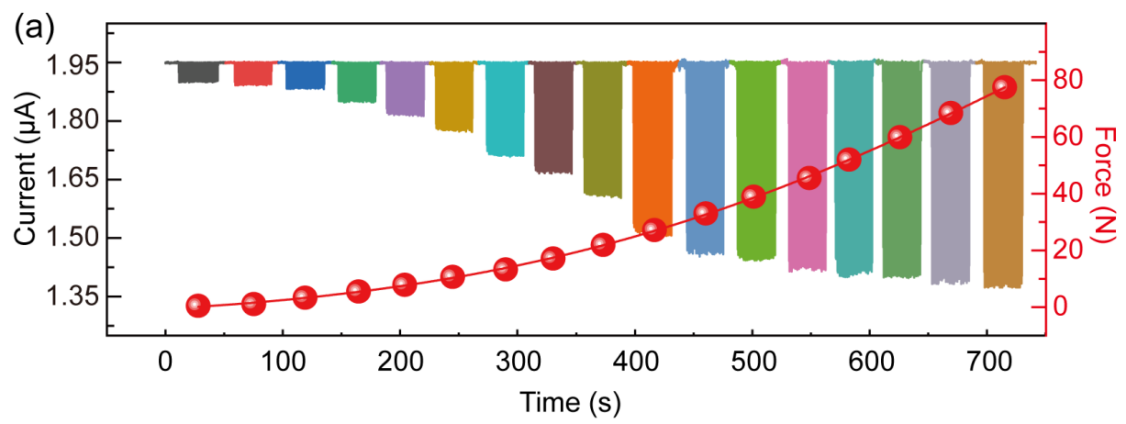


**Figure S8** The output curves of EOWS under different pressures.


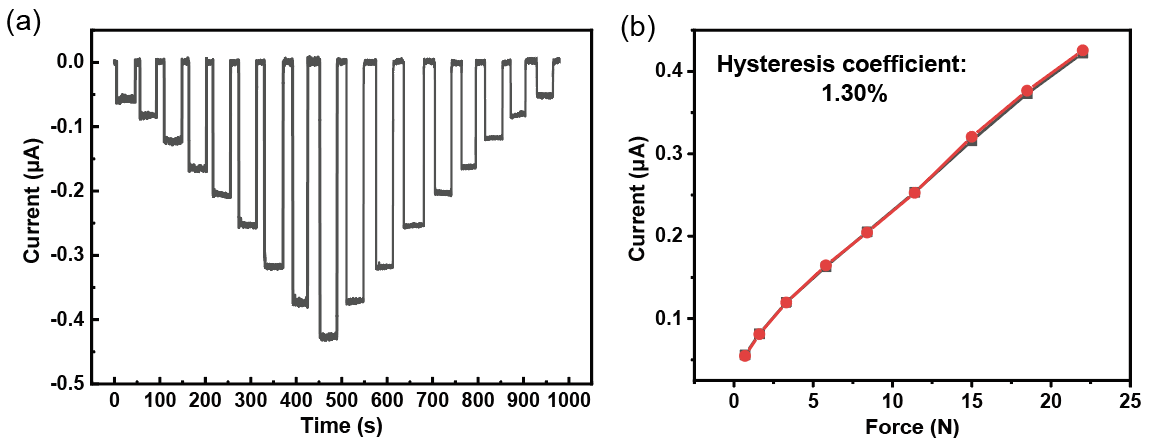


**Figure S9** (a) Output current curve of EOWS at different pressures. (b) Hysteresis curves of EOWS under different pressures.


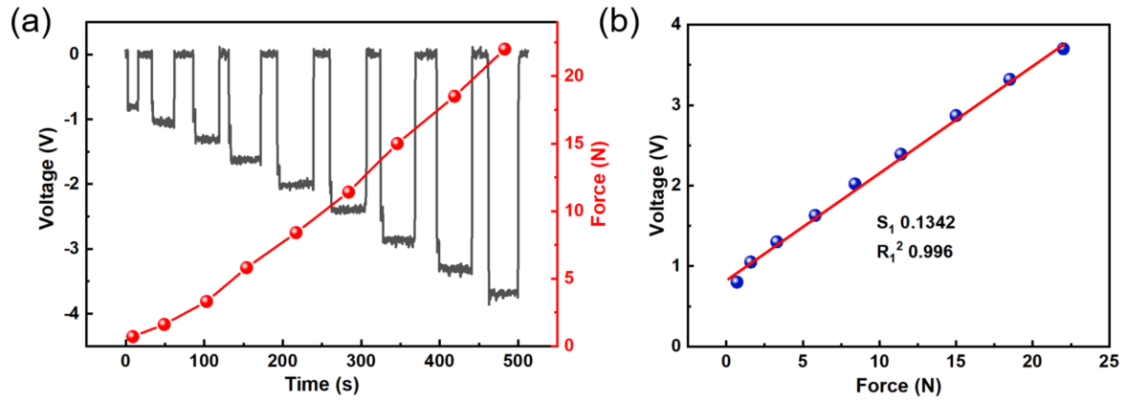


**Figure S10** (a) The output voltage curve of the inner surface EOWS under different pressures. (b) Voltage response of the inner surface EOWS under pressure of 0-22 N.


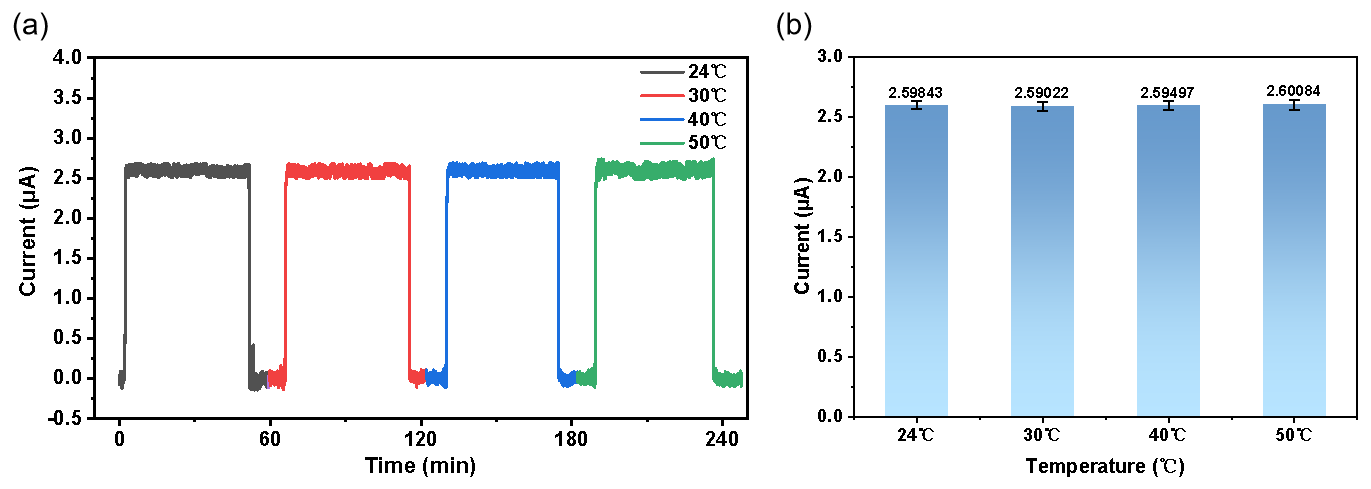


**Figure S11** (a) Current response of the EOWS under 24-50°C. (b) Mean value and standard deviation of the steady-state current.


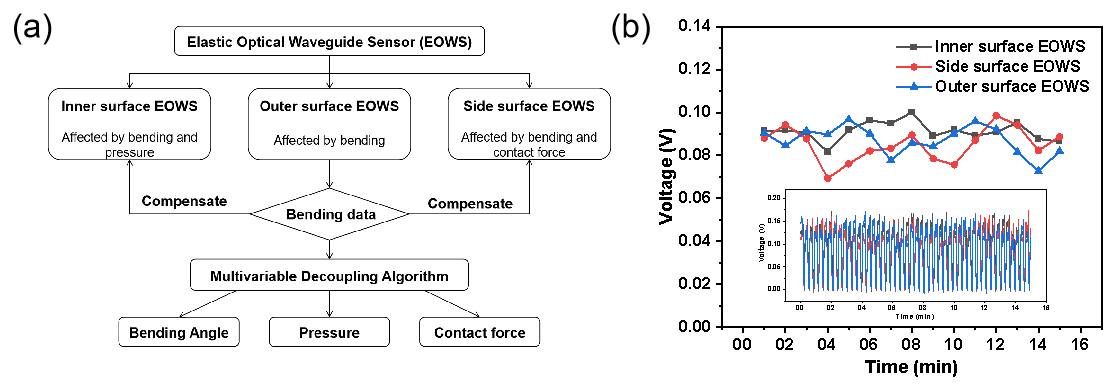


**Figure S12** (a) Schematic of the multivariable decoupling algorithm based on bending signal compensation. (b) The output response of the sensor in the empty grasping state.


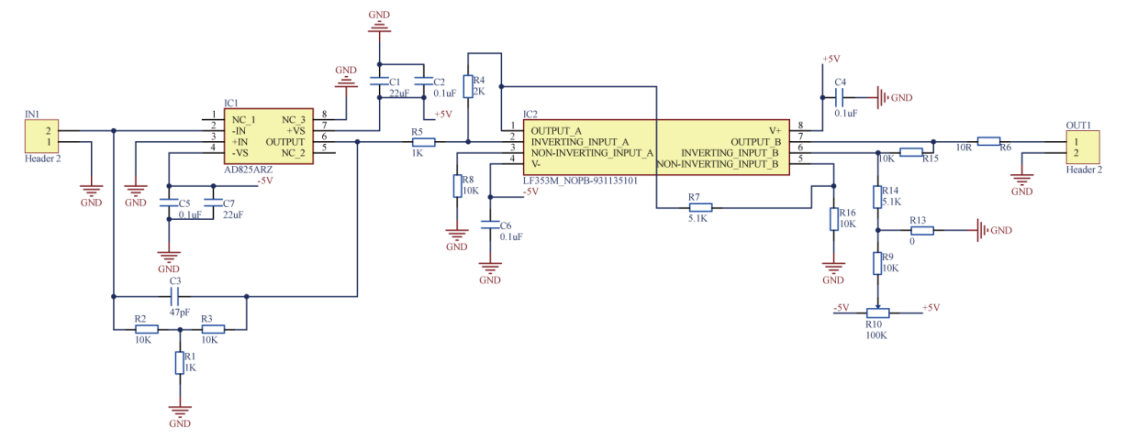


**Figure S13** Schematic diagram of 3-channel I-V transimpedance amplifier.


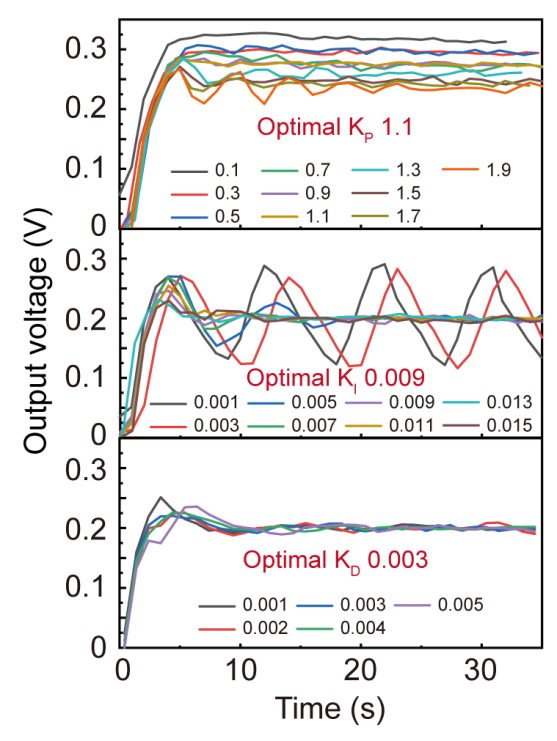


**Figure S14** Adjust the voltage response curves of K_P_, K_I_ and K_D_ under no-load conditions.


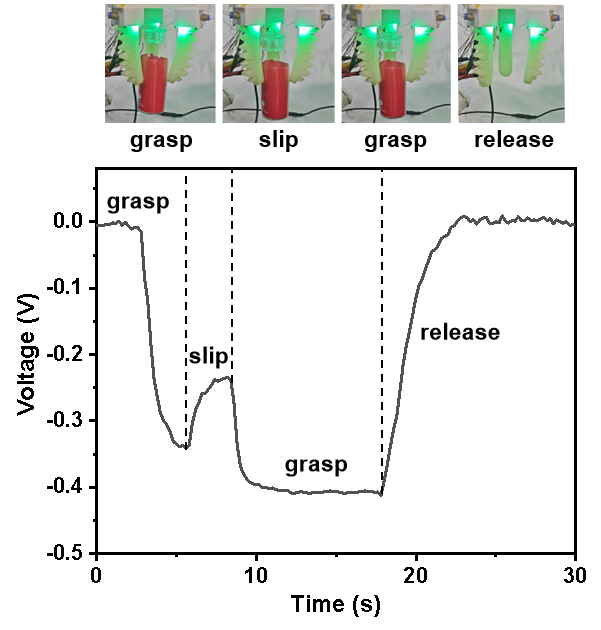


**Figure S15** Voltage response of soft robotic gripper in slip detection.


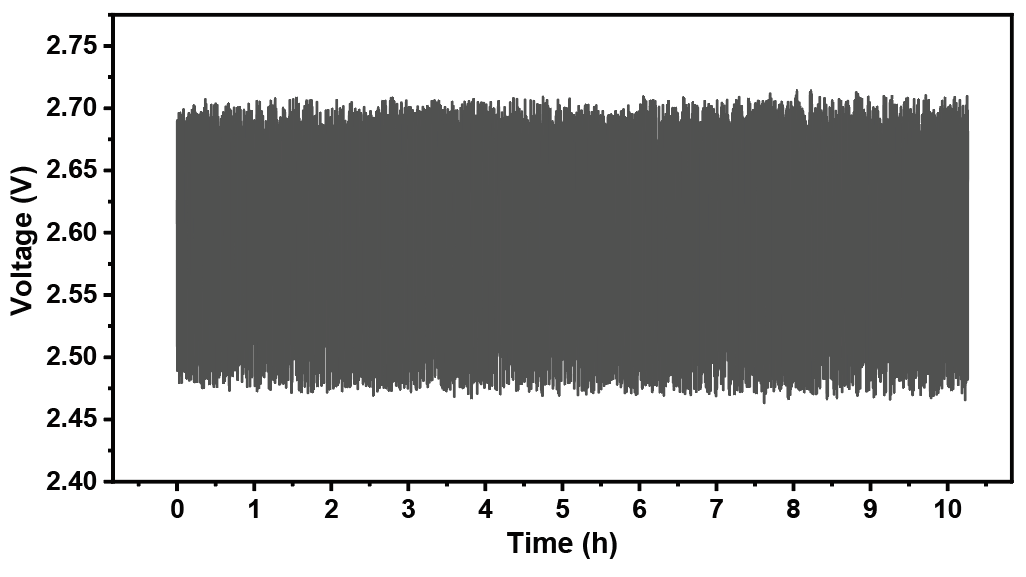


**Figure S16** Output stability of the soft robotic gripper during 10 h of continuous operation.


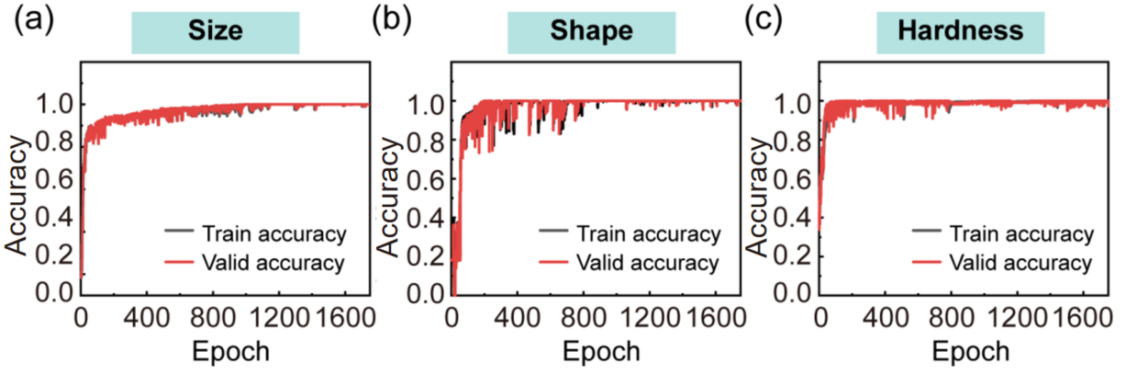


**Figure S17** The training efficiency curve of bio-inspired dexterous hands in recognizing the size, shape and hardness of objects.


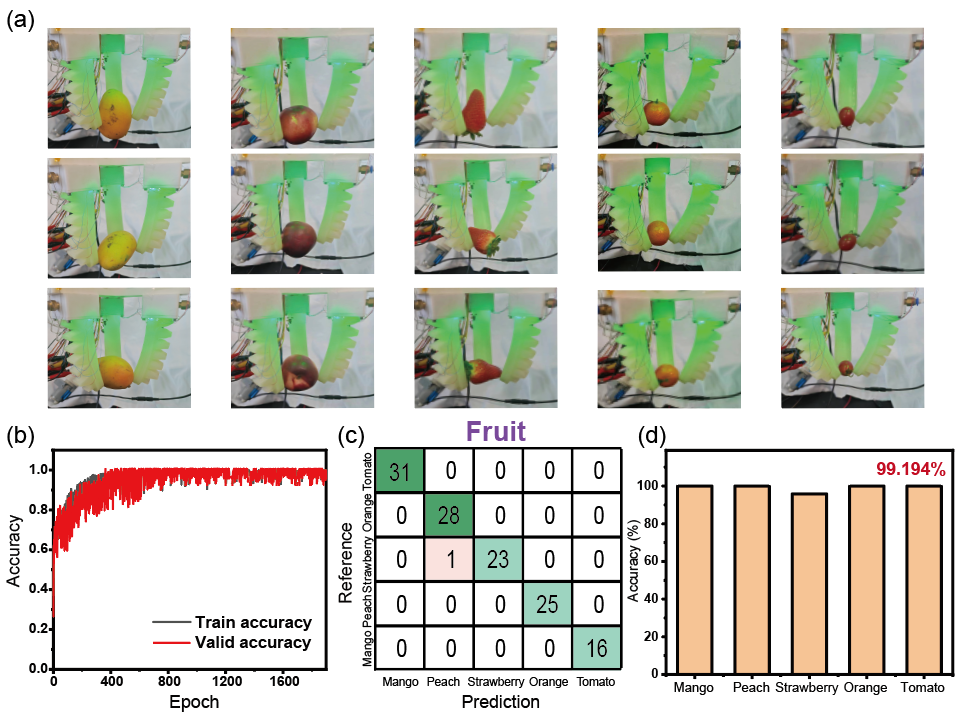


**Figure S18** (a) Soft robotic gripper grabs each fruit from different angles. (b-d) Training efficiency curve, confusion matrix, and recognition accuracy of soft robotic gripper recognition of fruit types (Fold 3).

**Table S1.** Comparison of the characteristics of different tactile sensing modalities.

| Sensing modality | Main advantages | Main limitations | Integration adaptability with soft robotic grippers | Robustness | Cost | Suitable tasks or scenarios |
| --- | --- | --- | --- | --- | --- | --- |
| Optical waveguide / optical tactile sensing | Electromagnetic-interference resistance; suitable for flexible embedding; capable of distributed, multimodal tactile information acquisition; beneficial for safe interaction and closed-loop control in soft robotic grippers | Output is affected by optical coupling, geometry, and loading mode; calibration, decoupling, and long-term consistency remain challenging | High. Soft robotic gripper tactile systems particularly require high compliance, conformal contact, small size, light weight, and high integration density; distributed integration has clear advantages | High (electromagnetic-interference resistance, no metal corrosion) | Low | Suitable for multimodal sensing of force/pressure/curvature/slip in soft robotic grippers, and for integration with closed-loop grasping control |
| Capacitive tactile sensing | High sensitivity and low power consumption; suitable for pressure/contact sensing; widely used in flexible electronics | Susceptible to noise and parasitic capacitance; signal instability may occur under large deformation and complex wiring; multimodal extension often requires more complex structures | Medium–high. Thin flexible structures are possible, but wiring and system redundancy increase complexity after array integration | Moderate (sensitive to electromagnetic environment and humidity) | Moderate | Suitable for contact detection, pressure distribution measurement, and flexible electronic skin |
| Piezoresistive tactile sensing | Relatively simple structure and readout circuit; easy to realize array integration; widely used in pressure and strain sensing | Often exhibits obvious hysteresis, drift, and repeatability problems; stability may be limited under large deformation or long-term operation | Medium–high. Flexible realization is relatively mature, but long-term consistency still requires attention | Moderate (sensitive to temperature and humidity) | Low | Suitable for pressure, strain, contact detection, and flexible electronic skin |
| Piezoelectric tactile sensing | Very fast dynamic response; self-powered (no external power supply); clear high-frequency signal | Unable to measure static force; fast charge leakage; relatively brittle materials (for inorganic piezoelectrics); limited bending radius | Medium. Polymer piezoelectric materials, such as PVDF, are flexible, but inorganic piezoelectric ceramics are difficult to conform to the large deformation of soft fingers | High (stable for dynamic signals, but no output for static force) | Moderate | Suitable for vibration detection, slip onset detection, dynamic touch, and impact recognition |
| Triboelectric tactile sensing | Self-powered; flexible structural design; high voltage output; capable of detecting contact/separation | Very high output impedance; signals are easily affected by environmental humidity and contamination; poor repeatability; difficult to calibrate | Medium–high. Can be made into thin films attached to the surface of soft robotic grippers, but requires a reliable contact–separation mode | Low (insufficient stability, easily affected by environmental disturbances) | Low | Suitable for self-powered touch sensing, material identification, and low-speed slip detection |
| MEMS / rigid pressure sensors | Mature quantitative calibration; well-defined output; good industrial availability and engineering translatability; relatively standardized readout | High rigidity; difficult to deeply integrate into highly compliant soft fingers; local stress concentration; requires flexible interconnection or isolation packaging | Medium–low. Requires rigid “islands” or elastomeric isolation packaging, which locally increases stiffness and may reduce overall compliance of the soft robotic gripper | High (the chip itself is reliable, but package interfaces may fail) | Relatively high | Suitable for scenarios requiring high quantitative accuracy and mature engineering solutions |
| Vision / camera-based tactile sensing | Capable of providing rich spatial contact information, such as morphology, texture, contact area, and slip; suitable for high-resolution contact perception | Larger system volume; depends on imaging and computation; affected by occlusion, field of view, illumination, and processing load | Medium. Can be used at the end effector of a soft robotic gripper, but is less direct than flexible tactile devices for compact, distributed embedding | Moderate (robustness is limited under complex visual conditions, and real-time computation cost is relatively high) | High | Suitable for high-resolution contact recognition, morphology reconstruction, and vision–tactile fusion |

**Table S2** The recognition accuracy rate of shape, size and hardness based on 5-fold cross-validation.

| **Fold** | **Shape accuracy (%)** | **Size accuracy (%)** | **Hardness accuracy (%)** | **Fruit type accuracy (%)** |
| --- | --- | --- | --- | --- |
| 1 | 97.824 | 100.00 | 97.713 | 98.925 |
| 2 | 97.915 | 100.00 | **97.987**​ | **99.053** |
| 3 | **98.077** | 100.00 | 97.802 | 99.194 |
| 4 | 97.786 | 100.00 | 97.695 | 98.876 |
| 5 | 97.853 | 100.00 | 97.751 | 99.012 |
| Mean | 97.891 | **100.00** | 97.790 | 99.012 |
| **Standard deviation** | 0.114 | **0.00** | 0.118 | 0.117 |

**Table S3** Performance of different sensors integrated into the soft robotic gripper.

| Types of sensors | Measurement | Sensitivity | Range | Attribute recognition | Recognition accuracy | Applications | References |
| --- | --- | --- | --- | --- | --- | --- | --- |
| Optical waveguide | Pressure  Bending angle  Contact force | 1.064%/N  0.273%/°  0.843%/N | 0-45 N  0-110°  0-15 N | shape, size, and hardness | >97% | Non-destructive grasping and attribute recognition | This work |
|  | Elongation  Curvature  Force | 2 dB/cm  0.02 dB∙m cm^-1^  / | 0-100%  20-70 m^-1^  0-20 N | Shape  Texture Softness | / | Object recognition | [1] |
| Piezoresistive | Bending angle | 0.444%/° | 0-90° | / | / | Gesture recognition | [2] |
|  | Pressure  Curvature | /  63.66%/cm^-1^ | 0-7.5 N  0-0.5 cm^-1^ | / | / | Soft grippers | [3] |
| Capacitive | Pressure | 0.0178 kPa^-1^ | 0-16 kPa | / | / | Fruit maturity grading | [4] |
|  | Pressure | 4.05 kPa^-1^ | 0-1 kPa | / | / | Soft gripper | [5] |
| Piezoelectric | Pressure  Curvature | 0.38 V kPa^-1^  0.12 mV m | 0-7  kPa  0-5.3 m^-1^ | / | / | Soft gripper | [6] |
|  | Pressure | 35.6 mV/N | 1-11 N | / | / | Monitor grasping conditions | [7] |
| Triboelectric | Pressure | / | 5-35 N | Fruit Recognition | 98.75% | A robotic hand | [8] |
|  | Bending degrees  Contact position and area | / | 0-180° | Object recognition | 98.1% | Soft gripper | [9] |

1. Zhao, H. C.; O'Brien, K.; Li, S.; Shepherd, R. F., Optoelectronically innervated soft prosthetic hand via stretchable optical waveguides. *Science Robotics* **2016,** 1, (1), eaai7529.

2. Dai, X. Y.; Wu, Y. H.; Liang, Q. H.; Yang, J. K.; Huang, L. B.; Kong, J.; Hao, J. H., Soft Robotic-Adapted Multimodal Sensors Derived from Entirely Intrinsic Self-Healing and Stretchable Cross-Linked Networks. *Advanced Functional Materials* **2023,** 33, (44), 2304415.

3. Meng, X. Y.; Tang, H. Y.; Lü, X. Z.; Shi, Y. G.; Bao, W. M., Electronic skin with shape sensing and Bending-Insensitive pressure sensing for soft robotic grippers object recognition. *Measurement* **2024,** 234, 114819.

4. Xia, J.; Wang, X. P.; Zhang, J. C.; Kong, C. Y.; Huang, W. T.; Zhang, X. S., Flexible Dual-Mechanism Pressure Sensor Based on Ag Nanowire Electrodes for Nondestructive Grading and Quality Monitoring of Fruits. *Acs Applied Nano Materials* **2022,** 5, (8), 10652-10662.

5. Su, M.; Fu, J. T.; Liu, Z. X.; Li, P.; Tai, G. J.; Wang, P. S.; Xie, L.; Liu, X. Q.; He, X. M.; Wei, D. P.; Yang, J., All-Fabric Capacitive Pressure Sensors with Piezoelectric Nanofibers for Wearable Electronics and Robotic Sensing. *Acs Applied Materials & Interfaces* **2023,** 15, (41), 48683-48694.

6. Xie, M. Y.; Zhu, M. Z.; Yang, Z. S.; Okada, S. N.; Kawamura, S., Flexible self-powered multifunctional sensor for stiffness-tunable soft robotic gripper by multimaterial 3D printing. *Nano Energy* **2021,** 79, 105438.

7. Huang, X. D.; Ma, Z. Y.; Xia, W. T.; Hao, L. X.; Wu, Y. H.; Lu, S.; Luo, Y. S.; Qin, L. G.; Dong, G. N., A high-sensitivity flexible piezoelectric tactile sensor utilizing an innovative rigid-in-soft structure. *Nano Energy* **2024,** 129, 110019.

8. Li, N.; Yin, Z. H.; Zhang, W. G.; Xing, C. Y.; Peng, T. J.; Meng, B.; Yang, J.; Peng, Z. C., A triboelectric-inductive hybrid tactile sensor for highly accurate object recognition. *Nano Energy* **2022,** 96, 107063.

9. Jin, T.; Sun, Z. D.; Li, L.; Zhang, Q.; Zhu, M. L.; Zhang, Z. X.; Yuan, G. J.; Chen, T.; Tian, Y. Z.; Hou, X. Y.; Lee, C., Triboelectric nanogenerator sensors for soft robotics aiming at digital twin applications. *Nature Communications* **2020,** 11, (1), 5381.
